# Genetic Evidence for Causal Relationships between Plasma Eicosanoid Levels and Cardiovascular Disease

**DOI:** 10.3390/metabo14060294

**Published:** 2024-05-23

**Authors:** Xukun Bi, Yiran Wang, Yangjun Lin, Meihui Wang, Xiaoting Li

**Affiliations:** 1 Key Laboratory of Cardiovascular Intervention and Regenerative Medicine of Zhejiang Province, Department of Cardiology, Sir Run Run Shaw Hospital, Zhejiang University School of Medicine, Hangzhou 310016, China; 2 Department of Nursing, No. 906 Hospital of People’s Liberation Army, Ningbo 315000, China

**Keywords:** eicosanoids, cardiovascular disease, genetic evidence

## Abstract

Cardiovascular diseases are the most common causes of mortality and disability worldwide. Eicosanoids are a group of bioactive metabolites that are mainly oxidized by arachidonic acid. Eicosanoids play a diverse role in cardiovascular diseases, with some exerting beneficial effects while others have detrimental consequences. However, a causal relationship between eicosanoid levels and cardiovascular disease remains unclear. Six single nucleotide polymorphisms (SNPs) with strong associations with plasma eicosanoid levels were selected. Summary-level data for cardiovascular disease were obtained from publicly available genome-wide association studies. A two-sample MR analysis identified that plasma eicosanoid levels were inversely correlated with unstable angina pectoris (OR 1.06; 95% CI 1–1.12; *p* = 0.04), myocardial infarction (OR 1.05; 95% CI 1.02–1.09; *p* = 0.005), ischemia stroke (OR 1.05; 95% CI 1–1.11; *p* = 0.047), transient ischemic attack (OR 1.03; 95% CI 1–1.07; *p* = 0.042), heart failure (OR 1.03; 95% CI 1.01–1.05; *p* = 0.011), and pulmonary embolism (OR 1.08; 95% CI 1.02–1.14; *p* = 1.69 × 10^−6^). In conclusion, our data strongly suggest a genetic causal link between high plasma eicosanoid levels and an increased cardiovascular disease risk. This study provides genetic evidence for treating cardiovascular diseases.

## 1. Introduction

Cardiovascular disease (CVD) has become a leading cause of mortality in the past few decades. In 2022, CVD will cause an estimated 19.8 million deaths, approximately one-third of all deaths globally, with ischemic heart disease and stroke accounting for 85% of all CVD deaths [1]. Recently, several modifiable risk factors have been recognized, including tobacco use, unhealthy diet, physical inactivity, and metabolic factors such as high blood pressure, high blood glucose, high cholesterol, and obesity. Although clinicians and researchers have made efforts to reduce the CVD burden by preventing or modifying these cardiovascular risk factors, the mortality rate associated with CVD remains high. Further elucidation of new therapeutic targets and the mechanisms underlying CVD is urgently required.

Eicosanoids are a diverse set of bioactive signaling lipids that are mainly derived from polyunsaturated fatty acids [2]. Arachidonic acid is a polyunsaturated fatty acid that constitutes the phospholipids in cell membranes. Free arachidonic acid is mainly liberated from cell membranes by cytoplasmic phospholipase (PLA2/PLC). De novo-synthesized arachidonic acid is derived from monounsaturated fatty acids, and stearoyl-CoA desaturase (SCD) is the rate-limiting enzyme that catalyzes the monounsaturated fatty acid synthesis [3]. Fatty acid desaturase (FADS) and the elongation of very long-chain fatty acids (ELOVL) also influence the arachidonic acid status by catalyzing the elongation of its precursors, linoleic acid and α-linolenic acid [4]. In addition, the activities of acyl-CoA thioesterase (ACOT) [5] and acetyl-CoA synthetase (ACS) [6] affect arachidonic acid levels through acyl-CoA metabolism. Arachidonic acid oxidation by cyclooxygenase (COX), lipoxygenase (LOX), and cytochrome P450 (CYP) pathways generates eicosanoids. In the COX pathway, arachidonic acid is converted to prostaglandin H2 (PGH2) by the peroxidase activity of COX, which is then sequentially metabolized to prostaglandins (PGs) and thromboxanes (TXs). The LOX pathway oxidizes arachidonic acid into several bioactive metabolites, including hydroxyeicosatetraenoic acids (HETEs) and leukotrienes (LTs). The CYP pathway metabolizes arachidonic acid to HETEs, hydroperoxyeicosatetraenoic acids (HPTETEs), and epoxyeicosatrienoic acids (EETs) [7]. Eicosanoids initiate various signaling cascades and regulate homeostatic processes. PGs and TXs can bind to cognate G protein-coupled receptors. Moreover, 5-LOX-derived LTs and intermediate metabolites, HPETEs and HETEs, serve as ligands for the peroxisome proliferator-activated receptors that regulate inflammation and lipid homeostasis [8].

Eicosanoids play diverse and partially contrasting roles in CVD pathogenesis. Eicosanoids are considered pro-inflammatory components. Studies on eicosanoids in inflammation have mainly focused on the signaling pathways activated by the metabolites generated by the COX pathway. Aspirin, a COX1 inhibitor, is a major treatment for coronary heart disease and ischemic stroke due to its anti-inflammatory and antithrombotic properties. COX1 inhibition suppresses the synthesis of eicosanoids, including PGs and TXs [9], and inhibits platelet aggregation and inflammation in atherosclerotic lesions. The prescription of aspirin lowers the incidence of all myocardial infarctions and major cardiovascular events by 36% and 15% [10], respectively. However, COX2 inhibition has been shown to increase the risk of CVD in several clinical studies. This negative effect is associated with hypertension exacerbation by inhibiting the production of the vasodilator autacoid PGI2, which is expressed by the vascular endothelium [11,12]. Interestingly, a growing body of research suggests that EETs derived from the CYP pathway exert protective effects against CVD [13]. Node et al. reported a new role of EETs as anti-inflammatory mediators [14]. The EETs are potent inhibitors of VCAM-1, ICAM-1, and E-selectin, induced by TNF-α, IL-1α, and bacterial lipopolysaccharide in human endothelial cells. This broad spectrum of anti-inflammatory effects is achieved through the suppression of the nuclear transcription factor κB pathway. This pathway is a common mechanism that mediates the stimulation of several cytokines. In addition, acetylcholine or bradykinin administration leads to arachidonic acid release from endothelial cells, which is sequentially metabolized by the CYP pathway to generate EETs [15]. EETs further stimulate smooth muscle cells to activate Ca^2+^-sensitive K^+^ channels, resulting in smooth muscle cell hyperpolarization and vasodilation [16]. Their anti-inflammatory and vascular relaxation properties make EETs a promising treatment target for hypertension and ischemic disease. In mice, the lack of a functional CYP gene and decreased EET levels increase systemic blood pressure [17,18]. Preclinical studies have also shown that ET analogs lower blood pressure, decrease kidney inflammation, and improve vascular endothelial function [19]. Altogether, the diverse functions of eicosanoids represent a barrier to identifying the relationship between total eicosanoids and CVD risk. However, a causal relationship between eicosanoid levels and CVD remains unclear.

This study aimed to provide genetic evidence on whether plasma eicosanoid levels affect the risk of multiple CVDs. We conducted a Mendelian randomization study by incorporating genetic variants of plasma eicosanoid levels into rational inference methods that diminished many typical biases and confounders as genetic variants independent of environmental factors. Since genetic variants are randomly assigned at conception, they are not affected by reverse causation. Thus, we employed a two-sample MR to evaluate the causal relationship between plasma eicosanoid levels and CVD.

## 2. Materials and Methods

### 2.1. Study Design

**Figure 1:**
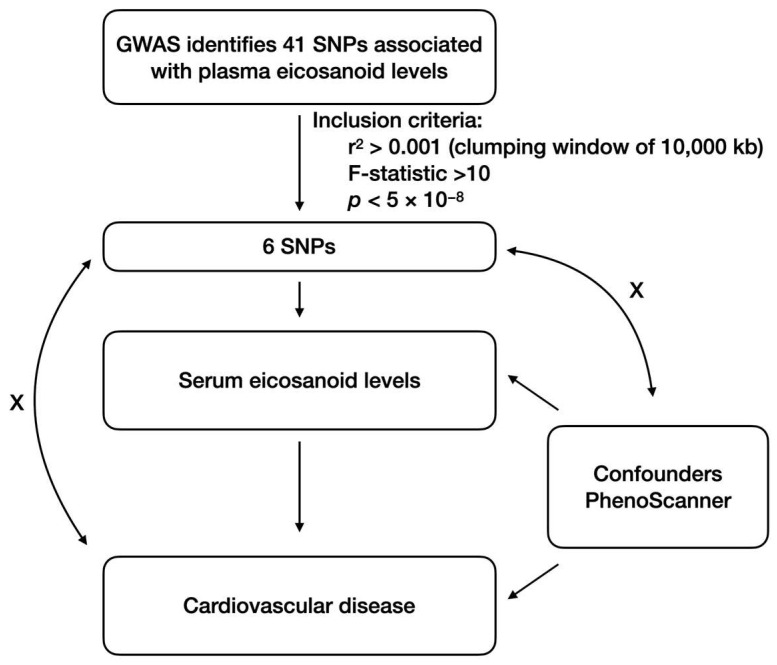
Graphical overview of the two-sample MR study design. The GWAS identified 41 single nucleotide polymorphisms (SNPs) associated with plasma eicosanoid levels. A series of quality control procedures were conducted to identify eligible genetic instruments. Firstly, SNPs were associated with plasma eicosanoid levels under a genome-wide significance threshold (*p* < 5 × 10^−8^). We used a clustering technique with r^2^ < 0.001 and a window size of 10,000 kb to avoid SNPs associated with significant linkage disequilibrium. Furthermore, all SNPs were cross-referenced using the PhenoScanner database v2 to verify their association with confounders and outcomes. Subsequently, we calculated the F-statistics of the SNPs for exposure. The F-statistic < 10 indicates the presence of weak instrumental bias. The arrows indicate the assumptions of MR analysis. Genetic instrumental variables are strongly associated with plasma eicosanoid levels but not related to confounding factors, and genetic instrumental variables should only be associated with the risk of CVDs via plasma eicosanoid levels. Abbreviations: GWAS, genome-wide association studies; SNPs, single nucleotide polymorphisms.

This study used a two-sample MR approach to evaluate the causal effect of plasma eicosanoid levels on CVD risk. The MR design is based on three core assumptions, as shown in Figure 1. There was a significant association between the genetic instrumental variables and plasma eicosanoid levels. Second, the absence of genetic instrumental variables was associated with confounding factors. Third, genetic instrumental variables should only be associated with the risk of CVD via plasma eicosanoid levels.

### 2.2. Data Sources

The analysis was conducted using published summary data from a GWAS on the plasma eicosanoid levels [20]. A total of 6496 participants of European ancestry from the Atherosclerosis Risk in Communities Study (ARIC) were included. In the overall sample, the mean age was 57.2 years; 52.9% were female, 21.1% were on treatment for hypertension, and 11.3% had diabetes. The mean estimated glomerular filtration rate was 98.6 mL/min/1.73 m^2^. Moreover, 33% were on aspirin medications, while 25.3% were on non-steroidal anti-inflammatory (NSAID) treatment. A total of 223 plasma eicosanoids and related metabolites were measured by directed non-targeted mass spectrometry using high-mass-accuracy liquid chromatography-mass spectrometry. In brief, plasma samples for eicosanoid profiling were collected and immediately stored at −80 °C. After both organic and solid-phase extraction, the samples were separated on a Phenomenex Kinetex C18 column by using mobile phases A (0.1% acetic acid, 30% acetonitrile, 70% water) and B (0.02% acetic acid, 50% acetonitrile, 50% isopropanol) with a gradient starting at 1% B to 99% B over 8 min. A Thermo Q Exactive orbitrap mass spectrometer was used to detect the mass.

Summary-level data for stable angina pectoris were obtained from a recent study conducted by Sakaue et al., which included 17,894 patients and 325,132 controls of European ancestry [21]. For unstable angina pectoris, GWAS summary data were obtained from the FinnGen Biobank consortium [22], a public research project in genomics and personalized medicine, which included 7058 cases and 197,630 controls. Summary statistics of myocardial infarction were obtained from the CARDIoGRAMplusC4D consortium [23], a combined database from multiple large-scale genetic studies to identify risk loci for coronary artery disease and myocardial infarction, including a total of 43,676 cases and 128,199 controls. Data on ischemic stroke were obtained from a study by Malik et al., with 7193 cases and 406,111 controls of European ancestry [24]. The GWAS summary data for heart failure (HF) were from Shah et al., including 47,309 cases and 930,014 controls [25]. Published data from Sakaue et al. were used for the aortic aneurysm analysis, with 3230 cases and 475,964 controls [21]. For atrial fibrillation and flutter, transient ischemic attack, hypertension, and pulmonary embolism, GWAS summary data were obtained from the FinnGen Biobank consortium. Supplementary Table S1 provides an overview of the datasets used in this study. All the GWAS outcome summary data were extracted from the IEU OpenGWAS project; therefore, no ethical approval was required. 

### 2.3. Selection of Genetic Instruments

The GWAS identified 41 genetic loci associated with plasma eicosanoid levels using a genome-wide threshold adjusted for the number of eicosanoids [20]. Single nucleotide polymorphisms (SNPs) were extracted from the GWAS summary data. A series of quality control procedures were conducted to identify eligible genetic instruments. To test the first hypothesis, SNPs were associated with plasma eicosanoid levels under a genome-wide significance threshold (*p* < 5 × 10^−8^) [26]. To ensure independence, we used a clustering technique with r^2^ < 0.001 and a window size of 10,000 kb to avoid SNPs associated with significant linkage disequilibrium. Furthermore, all SNPs were cross-referenced using the PhenoScanner database v2 to verify their association with confounders and outcomes. Subsequently, we calculated the F-statistics of the SNPs for exposure using the following formula: F = (R^2^/k)/([1 − R^2^]/[n − k − 1]), where R^2^ is the coefficient of determination, k is the number of instruments used in the model, and n is the sample size. The F-statistic < 10 indicates the presence of weak instrumental bias [27]. An overview of the selected genetic instruments is presented in Table 1.

### 2.4. MR Estimates

“Two sample MR” in the R package (version 0.5.10) was used to conduct MR analysis. MR-Egger [28], weighted median, inverse-variance weighted (IVW) [27], simple mode, and weighted mode methods [29] were used to estimate the effect value between the plasma eicosanoid levels and CVD. The IVW method was used for the primary analysis under random effects, which assumed all genetic variants were valid instrumental variances [30]. The MR-Egger and weighted median methods were used to assess the horizontal pleiotropy of selected instrumental variables [31]. The simple mode method gave a consistent estimate of the causal effect when at least 50% of the genetic variants were valid instrumental variables, regardless of the type of horizontal pleiotropy [29]. The weighted mode assumed that the most common causal effect was consistent with the true causal effect. Therefore, the remaining instruments could be invalid without biasing the estimated causal effect [32]. Statistical significance was set at a threshold of *p* < 0.05 and was used to determine the statistical significance. The results are presented as odds ratios (OR) with 95% confidence intervals (CI) in forest plots. 

### 2.5. Sensitivity Analysis

The sensitivity analysis aimed to identify possible heterogeneous and pleiotropic issues. Cochran’s Q test was used to evaluate heterogeneity using the MR-Egger and IVW methods. The values of each SNP and Cochran’s Q statistics are displayed in a forest plot. *p* < 0.05 was considered as evidence of heterogeneity [33]. A leave-one-out analysis was conducted to identify SNPs with non-proportional effects by removing one SNP from the analysis and re-estimating the causal effect. MR-Egger statistical analysis was used to limit the pleiotropic effect. The MR-Egger intercept test was used to investigate potential horizontal pleiotropic effects. A funnel plot was generated to visually inspect the potential pleiotropy. We also searched for SNPs in PhenoScanner to determine whether these SNPs were associated with secondary phenotypes [34].

## 3. Results

### 3.1. Characteristics of Selected Genetic Instruments

Initially, we identified 28 SNPs associated with plasma eicosanoid levels at the genome-wide significance level (*p* < 5 × 10^−8^) without significant linkage disequilibrium (Figure 1). Next, genetic instruments were removed if they were directly related to confounding factors based on the PhenoScanner database v2. We further deleted SNPs in the presence of weak instrumental bias if the F-statistics of these SNPs were below the threshold of 10. Finally, only 6 out of 28 SNPs were exclusively associated with plasma eicosanoid levels (Table 1).

Eicosanoids are derived from polyunsaturated fatty acids (PUFA). Among these six SNPs, rs174544 in the FADS1-3 locus and rs603424 in the PKD2L1 locus mainly participated in PUFA biosynthesis. rs111511359 in the ACOT4/ACOT6 locus and rs612490 in the ACSM6 locus are involved in fatty acyl-CoA metabolism, another distinct pathway in PUFA biosynthesis. Eicosanoid biosynthesis involves oxidation by enzymes, including cyclooxygenases, lipoxygenases, and cytochrome P450. rs4736317 in the CYP11B1/CYP11B2 locus is associated with cytochrome P450 loci. In addition, rs4149056 in the SLCO1B1 locus encodes a solute carrier organic anion transporter responsible for eicosanoid clearance.

### 3.2. Causal Effects of Plasma Eicosanoid Levels on CVD

**Figure 2:**
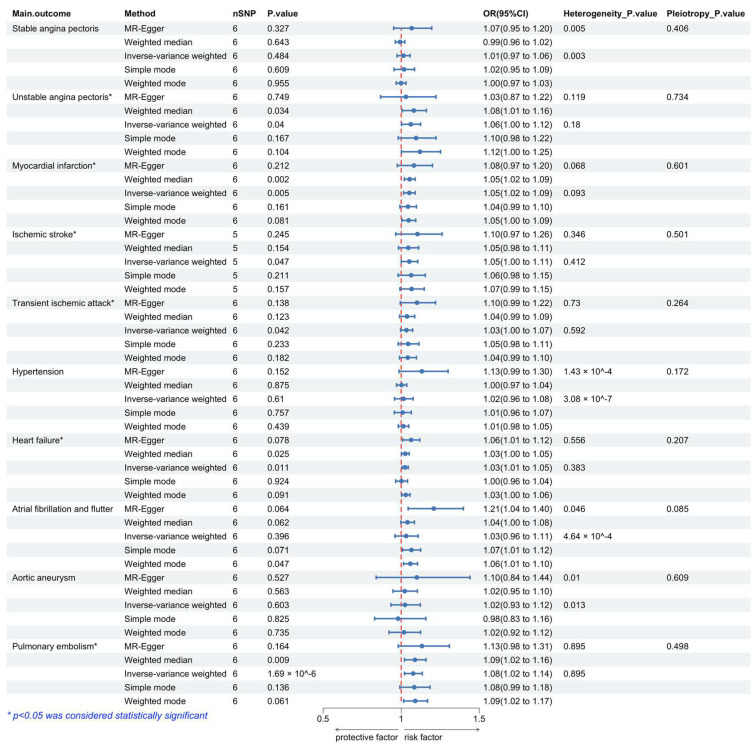
Associations between genetically predicted plasma eicosanoid levels and cardiovascular disease. Abbreviations: OR, odds ratio; CI, confidence interval.

Our MR analysis using the random-effects IVW method yielded results for plasma eicosanoid levels and CVD. The scatterplot in Supplementary Figure S1 compares the five MR analyses of different CVDs. The data are reported as odds ratios (OR) along with their corresponding 95% confidence intervals (CI), as shown in Figure 2. The results showed no genetic association between the plasma eicosanoid levels and stable angina pectoris (OR 1.01; 95% CI 0.97–1.06; *p* = 0.484). However, we found that unstable angina pectoris (OR 1.06; 95% CI 1–1.12; *p* = 0.04), myocardial infarction (OR 1.05; 95% CI 1.02–1.09; *p* = 0.005), ischemia stroke (OR 1.05; 95% CI 1–1.11; *p* = 0.047), and transient ischemic attack (OR 1.03; 95% CI 1–1.07; *p* = 0.042) have a significant association with plasma eicosanoid levels. This indicates that higher plasma eicosanoid levels are genetically associated with an increased risk of ischemic CVDs. Moreover, the high plasma eicosanoid levels also increase the risk of HF (OR 1.03; 95% CI 1.01–1.05; *p* = 0.011). Moreover, the genetically predicted eicosanoid levels had no casual relation with hypertension (OR 1.02; 95% CI 0.96–1.08; *p* = 0.61), atrial fibrillation and flutter (OR 1.03; 95% CI 0.96–1.11; *p* = 0.396), and aortic aneurysm (OR 1.02; 95% CI 0.93–1.12; *p* = 0.603), respectively. Interestingly, the result also provided strong genetic evidence for eicosanoid levels as a risk factor for pulmonary embolism (OR 1.08; 95% CI 1.02–1.14; *p* = 1.69 × 10^−6^).

### 3.3. Sensitivity Analyses Did Not Display an Indication of Unknown Pleiotropy

The presupposition of the MR study for causality inference was the absence of pleiotropic biases [35]. As shown in Figure 2, the MR-Egger approach did not provide evidence of pleiotropy in any of the main analyses (*p* > 0.05). Furthermore, the leave-one-out analysis showed that the MR results were not influenced by individual SNPs (Supplementary Figure S2). The PhennoScanner database was used to exclude known pleiotropic variants. Finally, Cochran’s Q statistic indicated no heterogeneity between the MR-Egger and IVW methods for unstable angina pectoris, myocardial infarction, ischemic stroke, transient ischemic attack, HF, or pulmonary embolism (*p* > 0.05). For stable angina pectoris, atrial fibrillation and flutter, hypertension, and aortic aneurysm (*p*-value of Cochran’s Q statistic < 0.05), a random effects model was used. 

## 4. Discussion

This two-sample MR study represents the first analysis of the genetic association between plasma eicosanoid levels and the risk of CVD. Our findings provide genetic evidence that high plasma eicosanoid levels increase the risk of CVDs, including ischemic CVD, HF, and pulmonary embolism. The selected SNPs were robust indicators of plasma eicosanoid levels, with the F-statistic of each SNP above a threshold of 10. Multiple sensitivity analyses did not indicate unknown pleiotropy.

### 4.1. Eicosanoid and Ischemic CVD

Ischemic CVD is primarily caused by a lack of blood flow, owing to atherosclerotic plaques and arterial inflammation. Our data showed that high plasma eicosanoid levels increased the risk of ischemic CVDs, including unstable angina pectoris, myocardial infarction, ischemic stroke, and transient ischemic attacks. The eicosanoid profile in patients with an ST-elevation myocardial infarction showed that the PGE2, PGD2, and TXA2 levels were significantly decreased, and EETs were increased 6 h after percutaneous coronary intervention (PCI) compared to the levels collected 30 min before PCI surgery [36]. TXs and PGs are the major prostanoids produced by the COX pathway that affect the cardiovascular system [37]. The COX metabolizes arachidonic acid to PGH2 and subsequently generates specific PGs, including PGE2, PGD2, and TXA2. Consistent with our findings, TXA2 contributes to the pathogenesis of various thrombotic diseases [38]. In animal studies, selective thromboxane receptor antagonists significantly attenuated the myocardial infarct size [39]. PGI2 production by COX2 exhibits a protective role in myocardial ischemia-reperfusion injury [40], and the balance between PGI2 and TXA2 controls myocardial homeostasis. In the LOX pathway, LTs mainly contribute to atherogenesis and promote ischemic CVD progression [41,42]. 5-LOX is abundantly expressed in atherosclerotic lesions, and the number of 5-LOX-expressed cells increases in advanced lesions [43]. The LT receptor antagonist decreased atherosclerosis in ApoE-knockout mice [44] and reduced the infarct size in a murine ischemia-reperfusion model [45].

EETs derived from the CYP pathway have shown beneficial effects in the preclinical model of coronary artery disease. CYP2J2 overexpression by adeno-associated virus injection increased the number of EETs and protected against HFD-induced atherosclerosis in ApoE-knockout mice [46]. Moreover, cardiomyocyte-specific CYP2J2 overexpression attenuates cardiac ischemia-reperfusion injury by activating mitochondrial ATP-sensitive K+ channels and the p42/p44 MAPK pathway [47]. CYP2J2 overexpression in the endothelium protects against myocardial infarction-induced cardiac remodeling by promoting angiogenesis [48]. Similarly, EET administration reduced the myocardial infarct size [49]. In contrast, 20-HETE, a primary eicosanoid derived from the CYP pathway, mediates androgen-induced hypertension. Upregulation of 20-HETE synthase in CYP4A12 transgenic mice increased the blood pressure by 40%, and a 20-HETE antagonist prevented this increase [50], indicating that 20-HETE, derived from the CYP pathway, participates in ischemic CVD by increasing blood pressure. The contradictory effects of eicosanoids are probably due to different reasons. The EETs activate several cell survival pathways, including the MAPK and PI3K/AKT pathways. Moreover, EETs also improved mitochondrial function and prevented oxidative stress [37]. However, 20-HETE reduces sodium transport by inhibiting Na^+^/K^+^ ATPase activity and potentiates the vasoconstrictors by enhancing calcium influx through the voltage-gated L-type calcium channels [51]. Collectively, despite the inconsistencies in the effects of eicosanoids on ischemic CVD, our genetic evidence suggests that eicosanoids may serve as risk factors weighted by contradictory effects. 

### 4.2. Eicosanoid and HF

HF is a complex clinical syndrome characterized by inadequate cardiac output due to cardiac abnormalities. Depending on the ejection fraction, HF can be divided into four categories: HF with reduced ejection fraction (HFrEF), preserved ejection fraction (HFpEF), mildly reduced ejection fraction (HFmrEF), and improved ejection fraction (HFimpEF) [52]. Recently, Lau et al. reported that 70 of 890 eicosanoids and related metabolites were associated with HFpEF status, and prostaglandin and linoleic acid derivatives were associated with greater odds of HFpEF [53]. In the LOX pathway, 12/15-LOX overexpression in cardiomyocytes results in systolic dysfunction and increased cardiac fibrosis, which is associated with macrophage infiltration [54]. However, genetic deletion of 12/15-LOX in post-myocardial infarction patients resulted in reduced 12-HETE and activated CYP-derived EETs and showed reduced cardiac rupture, improved cardiac function, and better survival [55]. 

CYP-derived EETs represent a promising therapeutic strategy for cardiac remodeling and dysfunction. At baseline, a pilot study of patients with acute decompensated HFrEF showed increased plasma 14,15-EET levels [56]. Cardiac CYP2J2 overexpression significantly attenuates Angiotensin II (Ang II)-induced cardiac hypertrophy. Mechanically, these cardioprotective effects were associated with peroxisome proliferator-activated receptor γ activation, reduced oxidative stress, and the inhibition of the NF-κB and TGF-β1/Smad pathways [57]. Similarly, by using the same transgenic mice with Ang II infusion, CYP2J2 overexpression improved the cardiac dysfunction and fibrotic response by reducing the activation of the α subunits of the G12 family G proteins/RhoA/Rho kinase cascade and elevation of the NO/cyclic guanosine monophosphate level in cardiac tissue [58]. In vitro, cardiac fibroblast activation, proliferation, migration, and collagen production, induced by Ang II, were inhibited by exogenous 11,12-EET through the Gα12/13/RhoA/ROCK pathway. Our findings indicate that pro- and anti-inflammatory eicosanoids contribute to HF and serve as potential therapeutic targets.

### 4.3. Eicosanoid and Pulmonary Embolism

Our MR analysis revealed a strong association between the eicosanoid levels and pulmonary embolism. Pulmonary embolism is characterized by the occlusion of blood flow in the pulmonary artery, mainly due to a thrombus traveling from the deep veins of the lower limb [59]. Venous stasis, local hypercoagulability, and endothelial injury are three factors that facilitate thrombus formation. Clinical studies have shown that COX-1 inhibitors protect against pulmonary embolism in inflammatory joint disease and that COX-2 inhibitors may be associated with an increased risk of PE [60]. Venous thromboembolism requires platelet activation, which leads to prostaglandin and TX release. The COX-1 pathway blockade inhibits TX synthesis, which reduces adverse responses to pulmonary embolism. In a canine model of pulmonary embolism, treatment with a cyclooxygenase inhibitor prevented pulmonary dead space and pulmonary vascular resistance [61]. In the LOX pathway, lipoxygenase products induce neutrophil activation and facilitate endothelial permeability following a thrombin-induced pulmonary microembolism [62]. In addition, coagulation and platelet activation can be promoted by 15-LOX/15-HETE signaling by regulating the generation of IL-6 and monocyte chemoattractant protein-1 [63]. Although CYP-derived EETs have multiple beneficial effects, their role in pulmonary embolism remains unclear. 

### 4.4. Limitation

Despite these significant findings, this study has several limitations. First, the F-statistical power was not very strong in selected SNPs due to the limited number of participants taking plasma eicosanoids in the Atherosclerosis Risk in Communities Study. Second, the study was limited to populations of European ancestry due to the availability of genetic data. Third, heterogeneity was observed in the MR analysis for stable angina pectoris, atrial fibrillation and flutter, hypertension, and aortic aneurysms, which could produce false-negative results. It is impossible to completely exclude potential pleiotropic factors that may lead to biased estimates. Fourth, the results of this study were based on the GWAS database analyses; therefore, larger prospective cohort studies are warranted to confirm the causality between plasma eicosanoid levels and CVD.

## 5. Conclusions

This two-sample MR study evaluated the causality between plasma eicosanoid levels and various CVDs. Our analysis identified plasma eicosanoid levels as risk factors for unstable angina pectoris (*p* = 0.04), myocardial infarction (*p* = 0.005), ischemic stroke (*p* = 0.047), transient ischemic attack (*p* = 0.042), HF (*p* = 0.011), and pulmonary embolism (*p* = 1.69 × 10^−6^). This study provides genetic evidence for plasma eicosanoid levels as potential biomarkers and therapeutic targets for CVDs. Large-scale research is required to investigate these causal relationships in populations from diverse ethnic backgrounds, as well as the potential underlying mechanisms.

## Figures and Tables

**Table 1:** The characteristics of all 6 SNPs and genetic associations with plasma eicosanoid levels.

Locus	SNP	Chromosome	Position	Effect_Allele	Other_ Allele	Beta	Se	*p*-Value	Eaf	F
FADS1-3	rs174544	11	61800281	C	A	0.54	0.018	2.76 × 10^−196^	0.29	65
SLCO1B1	rs4149056	12	21178615	T	C	0.54	0.023	5.7 × 10^−120^	0.16	25
PKD2L1	rs603424	10	100315722	G	A	−0.41	0.022	6.12 × 10^−74^	0.18	17
ACOT4/ACOT6	rs111511359	14	73610482	G	T	−0.34	0.021	7.68 × 10^−59^	0.21	15
ACSM6	rs612490	10	95215869	A	G	−0.23	0.018	1.92 × 10^−38^	0.57	14
CYP11B1/CYP11B2	rs4736317	8	142901337	A	G	−0.20	0.018	6.37 × 10^−30^	0.56	11

## Data Availability

The data that support the findings of this study are available from the corresponding author upon request. The data are not publicly available due to privacy.

## Supplementary Materials

The following supporting information can be downloaded at: https://www.mdpi.com/article/10.3390/metabo14060294/s1, Figure S1, Scatter plot of the association between plasma eicosanoid levels and cardiovascular disease using all 6 SNPs; Figure S2, Leave-one-out sensitivity analysis of the association between plasma eicosanoid levels and cardiovascular disease using all 6 SNPs; Table S1, Baseline characteristics of plasma eicosanoid level and cardiovascular disease.
