# Supplementary material for: Genetic Evidence for Causal Relationships between Plasma Eicosanoid Levels and Cardiovascular Disease
Source: Metabolites. 2024 May 23;14(6):294. doi: 10.3390/metabo14060294 (PMC11206149; doi:10.3390/metabo14060294)
Supplement: Supplementary file 1 [file metabolites-14-00294-s001.zip › Bi et Supplementary.pdf]

SUPPLEMENTAL MATERIALS

Genetic Evidence for Causal Relationships between Plasma Eicosanoid Levels and Cardiovascular Disease

Xukun Bi [1], Yiran Wang [2], Yangjun Lin [1], Meihui Wang [1] and Xiaoting Li [1,*]

[1] Key Laboratory of Cardiovascular Intervention and Regenerative Medicine of Zhejiang Province, Department of Cardiology, Sir Run Run Shaw Hospital, Zhejiang University School of Medicine, Hangzhou 310016, China

[2] Department of Nursing, No. 906 Hospital of People's Liberation Army, Ningbo 315000, China

* Correspondence: lixiaoting26@zju.edu.cn

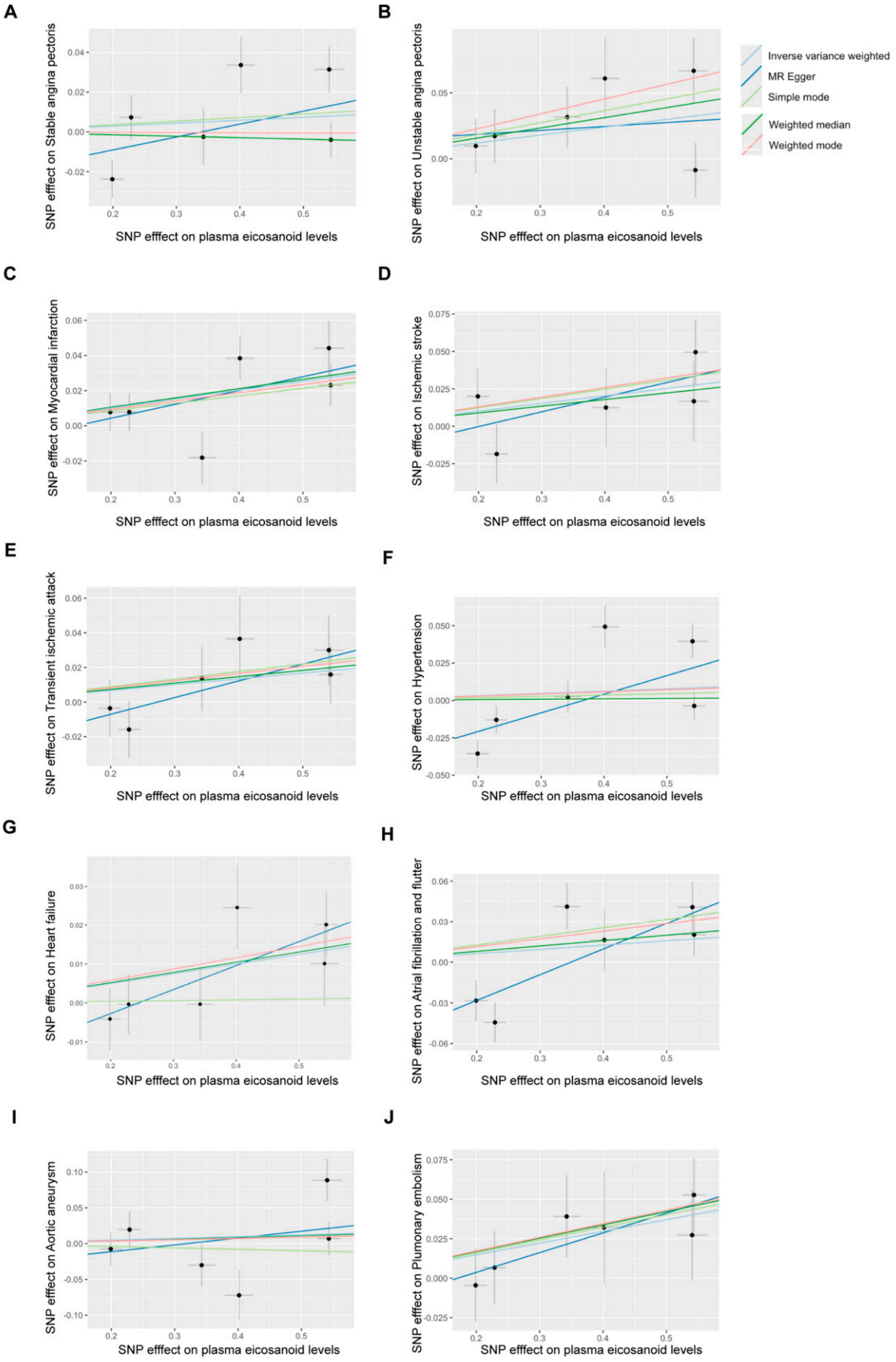

Supplementary Figure S1 Scatter plot of the association between plasma eicosanoid levels and cardiovascular disease using all 6 SNPs.

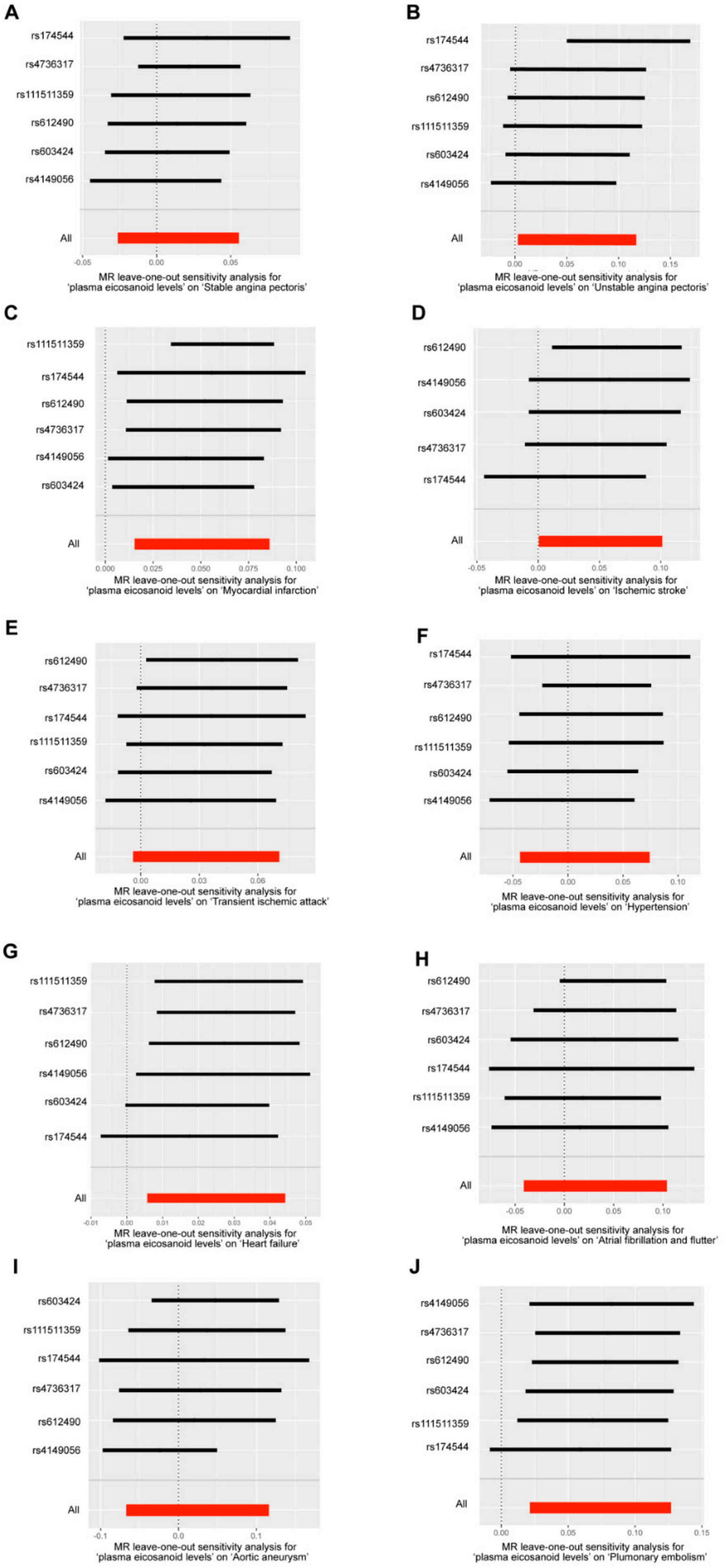

Supplementary Figure S2 Leave-one-out sensitivity analysis of the association between plasma eicosanoid levels and cardiovascular disease using all 6 SNPs.
