# Supplementary material for: Genetic Evidence for Causal Relationships between Plasma Eicosanoid Levels and Cardiovascular Disease
Source: Metabolites. 2024 May 23;14(6):294. doi: 10.3390/metabo14060294 (PMC11206149; doi:10.3390/metabo14060294)
Supplement: Supplementary file 1 [file metabolites-14-00294-s001.zip › Supplementary Table S1.pdf]

**Supplementary Table S1.** Baseline characteristics of plasma eicosanoid level and cardiovascular disease

| Trait | Year | Author/ Consortium | Population | Sample (n) | Case (n) | Control (n) | SNP (n) |
|---|---|---|---|---|---|---|---|
| Plasma eicosanoid levels | 2023 | Eugene P. Rhee | European | 6496 | - | - | 8,526,654 |
| Stable angina pectoris | 2021 | Saori Sakaue | European | 343,026 | 17,894 | 325,132 | 19,057,124 |
| Unstable angina pectoris | 2021 | FinnGen Biobank | European | - | 7,058 | 197,630 | 16,380,411 |
| Myocardial infarction | 2015 | CARDIoGRAMplusC4D | European (~ 77%) | 171,875 | 43,676 | 128,199 | 9,289,492 |
| Ischemic stroke | 2018 | Rainer Malik | European | 211,763 | 7,193 | 406,111 | 8,271,294 |
| Transient ischemic attack | 2021 | FinnGen Biobank | European | - | 8,835 | 202,223 | 16,380,430 |
| Hypertension | 2021 | FinnGen Biobank | European | - | 55,917 | 162,837 | 16,380,466 |
| Heart failure | 2020 | Sonia Shah | European | 977,323 | 47,309 | 930,014 | 7,773,021 |
| Atrial fibrillation and flutter | 2021 | FinnGen Biobank | European | - | 22,068 | 116,926 | 16,379,794 |
| Aortic aneurysm | 2021 | Saori Sakaue | European | 479,194 | 3,230 | 475,964 | 24,191,825 |
| Pulmonary embolism | 2021 | FinnGen Biobank | European | - | 4,185 | 214,228 | 16,380,466 |
